# Severe Mononeuritis Multiplex in Eosinophilic Granulomatous Polyangiitis: A Case Report

**DOI:** 10.7759/cureus.57283

**Published:** 2024-03-30

**Authors:** Reem Al Saadi, Sarah AlQassimi, Mohamed Abuzakouk, Ahmed Alduaij

**Affiliations:** 1 Internal Medicine, Cleveland Clinic Abu Dhabi, Abu Dhabi, ARE; 2 Allergy and Immunology, Cleveland Clinic Abu Dhabi, Abu Dhabi, ARE; 3 Pathology, National Reference Laboratory, Abu Dhabi, ARE; 4 Pathology and Laboratory Medicine Institute, Cleveland Clinic Abu Dhabi, Abu Dhabi, ARE

**Keywords:** monoclonal antibody, neuropathy, nucala, vasculitis, eosinophilic granulomatous polyangiitis, mepolizumab, mononeuritis multiplex

## Abstract

This report describes a 48-year-old man who presented with a month history of weakness and paraesthesia associated with severe pain of all four limbs. Initially diagnosed and treated as Guillain Barre syndrome due to the severity of his extremity weakness, it was later discovered to be eosinophilic granulomatous polyangiitis (EGPA).

Mononeuritis multiplex should not be underestimated or overlooked in the setting of diagnosing EGPA and requires prompt treatment with biologics to limit the permanent consequences on patient’s quality of life with regard to developing limb weakness and pain.

Although peripheral neuropathy, namely, mononeuritis multiplex, is not the most common feature of EGPA, it is important to consider it in order not to delay treatment with biologic agents that as seen in our patient can both halt the progress of the disease as well as give the patient a better quality of life.

## Introduction

Eosinophilic granulomatous polyangiitis (EGPA) is a rare yet potentially life-threatening necrotizing vasculitis affecting the small vessels. This disease develops over three phases: the prodromal/allergic phase (characterized by hard-to-treat asthma, sinusitis, and allergic rhinitis) then followed by the second eosinophilic stage with peripheral or organ infiltrating eosinophilia and the last stage being the vasculitic stage [1]. Not all patients develop the three; however, our patient did demonstrate all three phases with the last one being the most severe manifestation targeting the peripheral nerves. Vasculitic neuropathy occurs when inflammation affects the vasa nervorum that supplies nerves or the blood vessels surrounding the nerves (epineural precapillary vessels), causing reduced blood flow, tissue damage, and nerve dysfunction [1]. 

This article was previously posted to the Authorea preprint server on October 25, 2023.

## Case presentation

A 48-year-old gentleman with moderate-severe chronic rhinosinusitis and asthma initially presented to an outside hospital with a progressive four-week history of paresthesia and weakness of all four extremities, more prominently on the right compared to the left. He reports testing positive for COVID-19 infection one month prior, for which he was treated with a five-day course of favipiravir. After taking the first dose of medication, he started to develop paresthesia involving his right hand that spread to his forearm and then soon developed weakness and pain as well. The paresthesia and weakness progressed to involve the right leg, left arm, and left leg after a few days.

During his stay at the outside hospital, he was diagnosed with Guillain-Barre syndrome (GBS) based on a nerve conduction study that showed demyelinating motor neuropathy and conduction block of the right median, right ulnar, right tibial, and left peroneal nerves. Additionally, he underwent an electromyogram that confirmed the absence of motor nerve activity in a similar distribution. He was treated with IVIG and IV methylprednisone 500mg for five days with mild improvement and was discharged afterward. Unfortunately, his symptoms continued to worsen to the point he developed right wrist and bilateral foot drop, prompting his return to the hospital for further management.

He underwent further testing including laboratory work-up that revealed the following results (Table 1).

**Table 1:** Lab results pANCA: peri-neutrophil cytoplasmic antibodies; CRP: C-reactive protein; ESR: erythrocyte sedimentation rate

Lab	Result	Normal values
White blood count	19.7 x 10^9^/L	4.5 – 11.0 x10^9^/L
Neutrophils	8.9 x 10^9^/L	1.8 - 7.7 x10^9^/L
Eosinophils	7.23 x 10^9^/L	0.0 - 0.7 x10^9^/L
pANCA	>8.0	0.0 - 0.9 AI
IgE	1625 IU/mL	0 - 100 IU/mL
CRP	135 mg/L	0.0 - 4.9 mg/L
ESR	94 mm/hr	2 – 28 mm/hr
Creatinine	69 mmol/L	59 – 104 mmol/L
Complement C3	172 mg/dL	82 – 167 mg/dL
Complement C4	52 mg/dL	14 – 44 mg/dL

Other results included negative ANA, positive cryoglobulin IgG, and microscopic hematuria. He also underwent a lumbar puncture that yielded a white blood cell count of 2, elevated IgG, normal protein and a normal glucose level. Imaging included MRI spine which revealed a mild disc bulge at C3-C4 and C4-C5 and MRI brachial plexus which revealed no brachiopathy. He was started on oral prednisolone 60 mg daily and transferred from the outside hospital for evaluation of presumed eosinophilic granulomatosis with polyangiitis.

On admission to our facility, physical examination revealed 0/5 strength of the distal right upper extremity (right wrist drop), 5/5 of the proximal right upper extremity, 4/5 power of the distal left upper extremity, 5/5 power of the proximal left upper extremity, 0/5 power of the distal left lower extremity (left foot drop), 4/5 power of the right lower extremity, and marked loss of sensation in the lower extremities bilaterally up to the mid-shins.

Repeated extensive nerve conduction studies of the right upper and lower extremities and some additional studies of the left upper and lower extremities revealed significant asymmetrical widespread nerve conduction findings and concentric needle electrode findings consistent with vasculitic neuropathies, moderate to severe in degree electrically with axonal loss affecting the right upper and right lower extremities more than the left (Tables 2, 3). There was no electro-diagnostic evidence of an acute inflammatory demyelinating polyneuropathy or Guillan Barré Syndrome. 

**Table 2:** Sensory nerve conduction NR: No response

Sensory Nerve Conduction																	
Nerve	Recording	Stimulus	B-P Amp (μV)		Duration (ms)		LatNPk (ms)		CV (m/s)		Dist (mm)		Norm B-P Amp	Norm LatNPk	Norm CV	Temp	
			L	R	L	R	L	R	L	R	L	R				L	R
Median	Index	Wrist	8	NR	1.35	NR	3.2	NR	50	NR	130	130	>12	<3.4	>50	33.2	33.2
Ulnar	Dig V	Wrist	27	NR	1.20	NR	2.4	NR	57	NR	110	110	>12	<3.1	>50	33.4	33.2
Radial	Snuff Box	Forearm	28	NR	1.05	NR	1.88	NR	72	NR	100	100	>18	<2.7	-	33.5	33
Sural	Lat Mall	Mid Calf	2	NR	1.35	NR	3.1	NR	56	NR	140	140	>5	<4.5	>40	31.5	31.8
Superficial Peroneal	Ankle	14 cm	NR	NR	NR	NR	NR	NR	NR	NR	140	140	>5	<4.5	-	31.2	31.4

**Table 3:** Motor nerve conduction NR: No response

Motor Nerve Conduction																		
Nerve	Recording	Stimulus	B-P Amp (mV)		Duration (ms)		LatOn (ms)		CV (m/s)		Dist (mm)		Norm B-P Amp	Norm LatOn	Norm CV	Temp		
			L	R	L	R	L	R	L	R	L	R				L	R	
Median (ABP)	APB	Wrist	NR	NR	NR	NR	NR	NR			70	70	>6.0	<3.9		23.9	33	
Ulnar (ADM)	ADM	Wrist Bel Elbow Abv Elbow	8.6 8.1 7.5	NR	5.5 6.3 6.9	NR	2.3 6.1 8.1	NR	55 50		70 210 100	70	>7.0 - -	<3.1 - -	>50 >53	33.6 33.6 33.6	32.9	
Tibial (AHB)	AHB	Ankle Knee	1.67 1.08	NR	6.7 9.8	NR	3.9 9.8	NR	64		90 380	90	>8.0 -	<6.0 -	>40	31.5 31.5	30.8	
Peroneal (EDB) (EDB)	EDBl	Ankle Bel Fib Head Pop Fossa	0.52 0.30 0.30	NR	7.7 11.8 12.2	NR	4.4 11.5 13.6	NR	46 48		70 330 100	90	>3.0 - -	<5.5 - -	>40 -	31.4 31.4 31.4	31.5	
Peroneal (TA) (TA)	TA	Bel Fib Head Pop Fossa	1.05 1.03	0.32	15.4 14.7	-	2.9 4.6	20.5	59		- 100	-	- -	- -	-	31.5 31.5	31	

**Figure 1:**
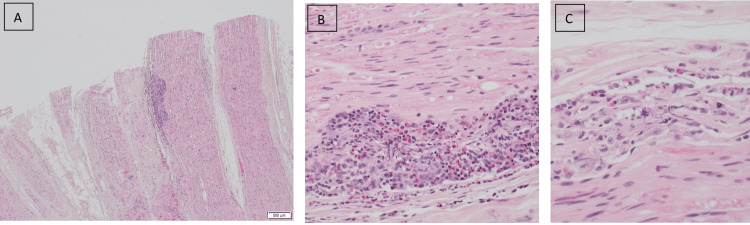
Histologic sections of the sural nerve showing (A&B) Occasional blood vessels show angiocentric inflammation marked by increased numbers of macrophages, lymphocytes, and eosinophils. (C) Occasional small collections of histiocytic cells are noted and may represent early granuloma formation. Hematoxylin and eosin (H&E) staining. Original magnification x40 (A), x200 (B), and ×400 (C)

He underwent nerve biopsy of the left sural nerve that revealed focal angiocentric acute and chronic inflammation (Figure 1).

He was promptly started on mepolizumab 300 mg subcutaneously with continuation of oral prednisolone 60 mg. His hospital stay was complicated by worsening severe neuropathic pain that required consultation with our pain management team. He was started on a pain regimen including oral pregabalin 150 mg twice daily, oxycodone 10 mg daily with OxyNorm 5 mg as needed, duloxetine 60 mg, and intravenous morphine 2 mg every three hours as needed. Further progression of his neurological deficits was evidenced by a new onset right foot drop. In view of his worsening symptoms, he was switched from mepolizumab to intravenous rituximab 1 g but there continued to be an increase of inflammatory markers It was felt that he was not responding to the treatment, and the decision to give two doses of IVIG with an increase of oral prednisolone from 60 mg to 90 mg.

After these changes, he showed both biochemical and clinical improvement and was discharged with close follow-up with Allergy and Immunology as an outpatient.

He was on mepolizumab for two months and tapering dose of prednisolone but due to financial constraints, he was unable to continue on mepolizumab. In view of his clinical improvement, he was started on azathioprine to maintain remission of the disease and his disease has been stable.

## Discussion

EGPA which is also called Churg-Strauss syndrome is an ANCA-related small vessel vasculitis presenting with a three-phase progressive course: a prodromal allergic phase of asthma and rhinosinusitis, then eosinophilic inflammatory infiltrates develop in the tissues and lastly a vasculitic phase such as purpura and mononeuritis multiplex. Pathophysiology of the latter is caused by axonal degeneration of the nerves due to ischemia caused by the vasculitis.

Although this neuropathic involvement is less likely to be detrimental compared to lung and renal involvement, it does markedly affect the patient’s quality of life and also predicts the need for add-on treatments [2]. Our patient presented with paraesthesia which then progressed to weakness of all four limbs and right wrist drop and bilateral foot drop in a manner not characteristic of GBS associated with severe neuropathic pain, with notable eosinophilia and a history of asthma, with no other organ involvement.

Mononeuritis multiplex and peripheral neuropathy are a typical presentation in patients diagnosed with EGPA. In a large published series, about 50-60% suffered from peripheral neuropathy and mononeuritis multiplex was slightly more common than peripheral neuropathy, with lower limbs being the most affected [3,4] with the peroneal, tibial, and ulnar being the most common nerves to be attacked, furthermore complicated by asymmetric foot or wrist drop, also symmetric or asymmetric polyneuropathy; sensory deficits and neuropathic pain [5,6] as was seen in our patient.

EGPA should always be in the differentials when thinking of treating or diagnosing a patient with GBS and a concurrent history of asthma as the treatments differ and may cause life-threatening complications. Accurately diagnosing EGPA is imperative due to the treatment delay-related progression into irreversible organ dysfunction.

Management of EGPA initially requires the use of high-dose systemic corticosteroids with the aim to achieve and maintain clinical remission swiftly. Second line, in cases that do not respond to steroids, other immunosuppressants like rituximab and cyclophosphamide are needed. Other promising treatment options are biologic agents, such as anti-interleukin-5 mepolizumab which was used in our patient. Prognosis of untreated EGPA is very poor with a reported five-year survival of 25%. Whereas when diagnosed quickly and treated appropriately, prognosis is much improved, with a 78-month survival rate of about 70-90% [7]. Mepolizumab has been effectively used in combination with prednisolone to treat peripheral neuropathy associated with EGPA and most recently, add-on low-dose mepolizumab showed good results for long-lasting neuropathy complications from EGPA [8]. 

## Conclusions

In conclusion, this case report highlights the importance of considering EGPA in the differential diagnosis of patients presenting with mononeuritis multiplex, especially in the context of a history of asthma or other allergic manifestations. The case underscores the challenges in diagnosing EGPA, particularly when initial symptoms mimic GBS. Early initiation of appropriate therapy is crucial in preventing irreversible organ damage and improving patient outcomes. Mononeuritis multiplex as a presentation of EGPA could foretell the need for aggressive treatment with biological agents like Mepolizumab, making peripheral neuropathy an important symptom to associate with EGPA and to guide management after diagnosis.

Furthermore, this case highlights the importance of a multidisciplinary approach involving specialists in immunology, neurology, pathology, and pain management for optimal patient care. Close monitoring and long-term follow-up are essential to ensure disease remission and prevent relapse. The case also emphasizes the critical role of early recognition, accurate diagnosis, and timely intervention in improving the prognosis and quality of life for patients with EGPA associated with severe mononeuritis multiplex.
